# Vitamin D in Digestive Neoplasms: Molecular Mechanisms and Clinical Evidence

**DOI:** 10.3390/ijms27209343

**Published:** 2026-10-21

**Authors:** Andreea-Ramona Treteanu, Ana Maria Alexandra Stănescu, Alexandru Constantinescu, Ovidiu Lucian Băjenaru, Cătălina Raluca Nuță, Gratiela Gradisteanu Pircalabioru, Octavian Andronic

**Affiliations:** 1 Faculty of Medicine, Carol Davila University of Medicine and Pharmacy, 020021 Bucharest, Romania; andreea-ramona.treteanu0720@stud.umfcd.ro (A.-R.T.); alexandra.stanescu@umfcd.ro (A.M.A.S.); ovidiu.bajenaru@umfcd.ro (O.L.B.); catalina.nuta@umfcd.ro (C.R.N.); octavian.andronic@umfcd.ro (O.A.); 2 Center of Innovation and eHealth, Carol Davila University of Medicine and Pharmacy, 020021 Bucharest, Romania; 3 Romanian Academy of Scientists, 030167 Bucharest, Romania; 4 Gastroenterology Department, University Emergency Hospital, 050098 Bucharest, Romania; 5 Ana Aslan National Institute of Gerontology and Geriatrics, 011241 Bucharest, Romania; 6 Department of Botany and Microbiology, Faculty of Biology, University of Bucharest, 030018 Bucharest, Romania; gratiela.gradisteanu@icub.unibuc.ro; 7 Research Institute of University of Bucharest (ICUB), University of Bucharest, 050095 Bucharest, Romania; 8 eBio-Hub Centre of Excellence in Bioengineering, National University of Science and Technology Politehnica Bucharest, 060042 Bucharest, Romania

**Keywords:** vitamin D, digestive neoplasm, cancer prevention

## Abstract

Digestive neoplasms represent a major global health burden, accounting for a substantial proportion of cancer incidence and mortality. Increasing attention has been directed toward modifiable factors that may influence both the onset and progression of these malignancies. Among them, vitamin D has emerged as a potential protective factor, with deficiency frequently associated with elevated risk and unfavorable prognosis in gastrointestinal cancers. This review critically examines the current evidence regarding the role of vitamin D and its receptor (VDR) in digestive tumorigenesis. Beyond its classical function in calcium and bone homeostasis, vitamin D exerts pleiotropic effects on cellular processes fundamental to carcinogenesis, including regulation of proliferation, differentiation, apoptosis, angiogenesis, immune modulation and genomic stability. Experimental studies demonstrate that adequate vitamin D status or administration of its analogues may suppress tumor growth and enhance responsiveness to conventional therapies. Epidemiological and clinical data, however, remain inconsistent across cancer subtypes, geographic populations and methods of vitamin D assessment. The analysis underscores the need for standardized evaluation of vitamin D levels, elucidation of dose–response relationships and clarification of interactions with genetic polymorphisms and environmental exposures. Future large-scale, randomized controlled trials are essential to establish causality and to define the optimal role of vitamin D in prevention and treatment strategies. Taken together, current findings support the hypothesis that vitamin D may serve not only as a biomarker of prognosis but also as a promising adjunctive approach in the management of digestive neoplasms.

## 1. Introduction

Digestive neoplasms are one of the major medical challenges of the 21st century, with an incidence of 1 in 4 diagnosed cancer cases and a mortality of 1 in 3 cancer deaths worldwide [1]. In the absence of early diagnosis, digestive cancers are associated with a poor prognosis and contribute to a significant decrease in patients’ quality of life.

The complexity of their etiology arises from the interplay of genetic, immunological and environmental factors, with recent research highlighting the multifactorial nature of these malignancies and the importance of modifiable risk factors in their development and progression. Among these, nutritional deficiencies, particularly vitamin D deficiency, have garnered increasing attention due to their potential role in both the risk and prognosis of digestive cancers [2].

Vitamin D is now recognized not only for its classical role in bone metabolism but also for its crucial immunomodulatory and anti-inflammatory effects, which are mediated through vitamin D receptors (VDRs) expressed on immune and intestinal epithelial cells. Deficiency in vitamin D has been associated with increased incidence and poorer outcomes in colorectal and other digestive cancers, likely due to its influence on immune surveillance, inflammation and the integrity of the gut barrier [3,4,5]. Furthermore, vitamin D status can modulate the gut microbiome, which in turn affects cancer immunity and response to therapies [6,7]. These findings underscore the importance of understanding vitamin D’s multifaceted role in digestive neoplasms and suggest that maintaining optimal vitamin D levels may offer a promising avenue for prevention and improved management of these cancers.

Several recent reviews have highlighted the expanding role of vitamin D and vitamin D receptor (VDR) signaling in gastrointestinal health and cancer. Sonsalla et al. provided a broad overview of vitamin D in gastrointestinal physiology and disease, emphasizing its involvement in epithelial barrier integrity, mucosal immunity, inflammatory regulation, and gastrointestinal homeostasis [8]. Other reviews have examined VDR signaling from a broader oncological perspective, addressing its biological functions, molecular mechanisms, prognostic relevance, and therapeutic potential across multiple malignancies [9,10]. More focused reviews have addressed the relationship between vitamin D status and pancreatic cancer risk and outcomes, as well as alterations in vitamin D metabolism and VDR signaling in hepatocellular carcinoma [11,12]. In addition, Khan et al. recently reviewed the role of vitamin D in gastrointestinal cancer prevention and management, integrating mechanistic, epidemiological, and interventional evidence and highlighting persistent uncertainties regarding its clinical translation [13].

Nevertheless, important aspects of the relationship between vitamin D and digestive neoplasms remain insufficiently integrated. Existing reviews have predominantly approached the subject from broad gastrointestinal, pan-cancer, mechanistic, or individual tumor perspectives, while a comparative tumor-specific synthesis across the major digestive malignancies remains limited. The present review addresses this gap by examining esophageal, gastric, hepatic, pancreatic, and colorectal cancers individually and integrating, for each malignancy, the available mechanistic, experimental, epidemiological, prognostic, and interventional evidence. This tumor-specific approach enables differences in the extent and strength of the available evidence to be identified across digestive cancer types and allows areas of consistency between biological and clinical findings to be distinguished from those in which evidence remains conflicting or predominantly preclinical. The review also delineates the major unresolved questions within each malignancy, thereby identifying tumor-specific priorities for future mechanistic, translational, and clinical research.

Accordingly, the purpose of this review is to provide a comprehensive and critical synthesis of the current evidence regarding the implications of vitamin D in digestive neoplasms, with particular emphasis on its biological mechanisms, prognostic significance, and potential therapeutic applications. Specifically, the objectives are to: (i) elucidate the molecular pathways through which vitamin D and VDR signaling may influence tumorigenesis; (ii) critically examine the experimental, epidemiological, and clinical evidence across individual digestive malignancies; and (iii) identify tumor-specific knowledge gaps and future research directions needed to clarify causality and facilitate clinical translation.

## 2. Review Methodology

This study was conducted as a narrative review to provide a comprehensive and critical synthesis of the evidence regarding the role of vitamin D and vitamin D receptor (VDR) signaling in the development, progression, prognosis, and potential therapeutic modulation of digestive neoplasms. A narrative approach was considered appropriate because the available literature encompasses substantial methodological and biological heterogeneity, including molecular and cellular studies, animal models, observational and epidemiological studies, clinical trials, systematic reviews, and meta-analyses. This design enabled the integration of mechanistic evidence with preclinical and clinical findings across different digestive malignancies.

A structured literature search was performed in PubMed, Scopus, and Web of Science using combinations of terms related to vitamin D biology and digestive neoplasms. The following search formula was used and adapted to the indexing system and search syntax of each database:

(vitamin D OR 25-hydroxyvitamin D OR 25(OH)D OR 1,25-dihydroxyvitamin D OR 1,25(OH)2D OR calcitriol OR cholecalciferol OR ergocalciferol OR vitamin D receptor OR VDR) AND (digestive cancer OR digestive neoplasm OR gastrointestinal cancer OR gastrointestinal neoplasm OR esophageal cancer OR oesophageal cancer OR gastric cancer OR stomach cancer OR liver cancer OR hepatocellular carcinoma OR cholangiocarcinoma OR biliary tract cancer OR pancreatic cancer OR colorectal cancer OR colon cancer OR rectal cancer).

No restrictions on publication date were applied, as earlier experimental and epidemiological studies were considered relevant for establishing the biological and conceptual foundations of vitamin D–related carcinogenesis. The database search was supplemented by backward snowball searching of the reference lists of relevant articles and reviews to identify additional studies of mechanistic or historical importance.

Studies were considered eligible when they addressed at least one aspect relevant to the objectives of this review, including vitamin D synthesis and metabolism; VDR expression or signaling; molecular mechanisms potentially involved in carcinogenesis; associations between vitamin D status and digestive cancer risk, progression, recurrence, survival, or prognosis; or the effects of vitamin D supplementation, calcitriol, or vitamin D analogues in experimental or clinical settings. Particular attention was given to studies investigating esophageal, gastric, hepatic, pancreatic and colorectal malignancies, reflecting the organ-based structure of the review. The manuscript itself follows this tumor-location organization to account for organ-specific biological characteristics and differences in VDR expression.

Original experimental studies, including in vitro and in vivo investigations, observational and epidemiological studies, prospective and retrospective cohort studies, case-control studies, and randomized controlled trials were considered. Systematic reviews and meta-analyses were additionally included when they provided higher-level evidence regarding associations between vitamin D status and cancer risk or clinical outcomes or facilitated interpretation of heterogeneous findings across individual studies. Relevant narrative reviews were used primarily to provide biological context and identify established or emerging mechanistic concepts.

Only peer-reviewed publications written in English were considered. Conference abstracts, letters, commentaries, preprints, dissertations, and other non-peer-reviewed publications were excluded. Studies without clear relevance to vitamin D/VDR biology or digestive neoplasms were also excluded.

Study selection was performed in two stages. First, titles and abstracts were screened for relevance to the scope of the review. Articles considered potentially eligible were then assessed by full-text review, and studies meeting the eligibility criteria were included in the final synthesis.

Retrieved studies were evaluated qualitatively according to several characteristics, including study design; type of digestive neoplasm; study population or experimental model; vitamin D status, exposure, or intervention; VDR-related mechanisms; and reported biological or clinical outcomes.

Rather than performing a formal risk-of-bias assessment or quantitative meta-analysis, evidence was synthesized narratively by comparing findings across digestive cancer types, identifying areas of agreement and disagreement across studies, and considering differences between preclinical, observational, and interventional evidence.

As a narrative review, this work has inherent methodological limitations. Unlike systematic reviews, study selection and evidence synthesis were not conducted using a prospectively registered protocol, duplicate independent screening, or formal risk-of-bias assessment. Consequently, some degree of selection bias cannot be excluded. Nevertheless, the structured search strategy, use of multiple bibliographic databases, clearly defined eligibility criteria, inclusion of high-quality systematic reviews and meta-analyses, and supplementary snowball searching were intended to maximize literature coverage and provide a balanced synthesis of the available evidence.

## 3. Physiology of Vitamin D and the VDR 

**Figure 1:**
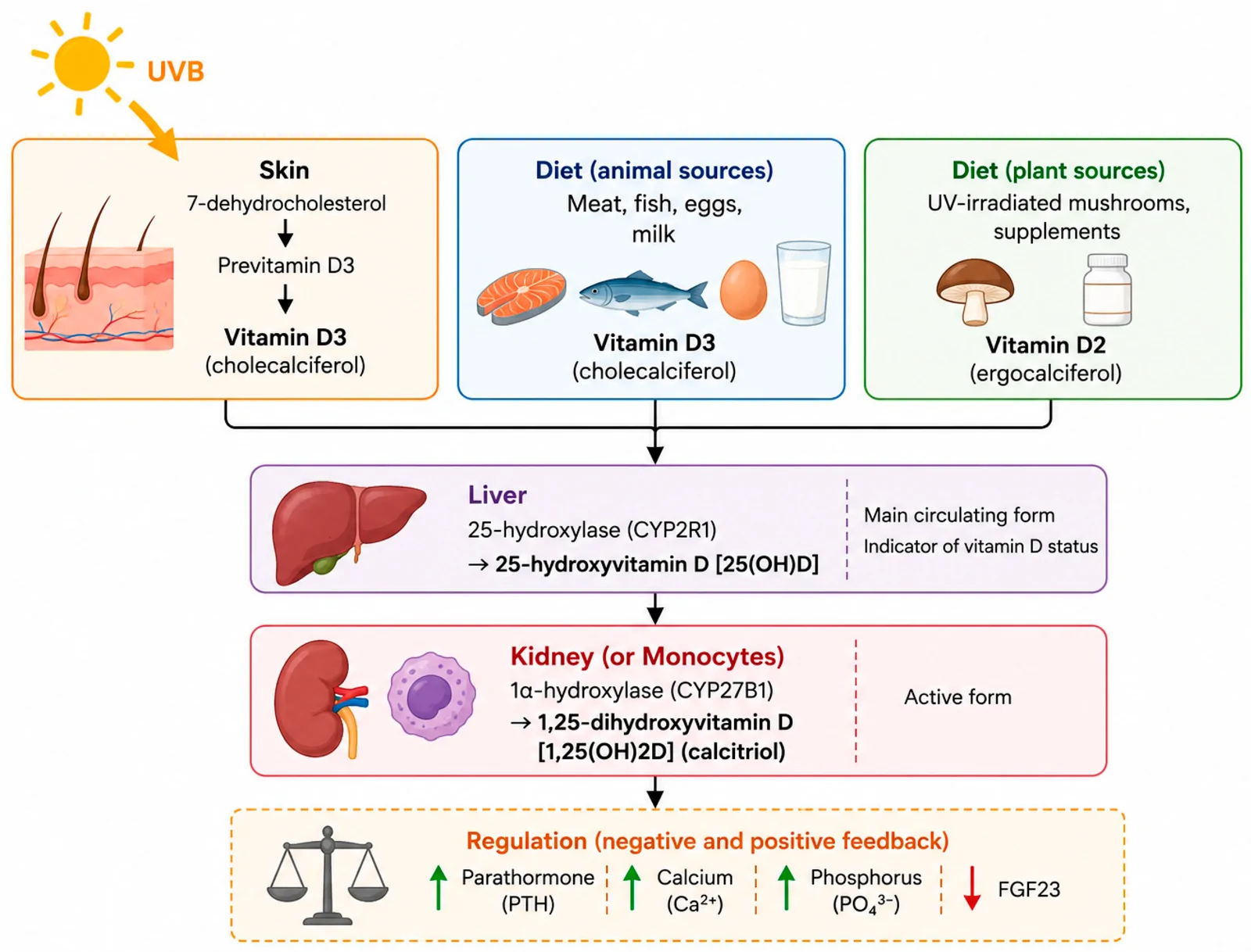
Schematic overview of vitamin D sources, synthesis, metabolism and activation.

A fat-soluble vitamin par excellence, vitamin D is essential for numerous biological functions, and its synthesis and metabolism involve several complex pathways. There are two primary forms of vitamin D: vitamin D2 (ergocalciferol) and vitamin D3 (cholecalciferol) [14]. The synthesis of vitamin D3 begins in the skin (Figure 1), where 7-dehydrocholesterol is converted to previtamin D3 under the action of ultraviolet B (UVB) radiation. Previtamin D3 is then isomerized to vitamin D3 (cholecalciferol). Vitamin D3 can also be obtained from meat, fish, eggs and milk [15]. Alternatively, vitamin D2 (ergocalciferol) is derived from plant sources such as UV-irradiated mushrooms and food supplements [16]. Both forms of vitamin D are physiologically inert. After its formation in the skin or its ingestion from food, vitamin D is transported to the liver, where it undergoes hydroxylation at position 25, resulting in 25-hydroxyvitamin D [25(OH)D], which is the main circulating metabolite and the standard indicator of vitamin D status in the body. In general, the normal range for circulating 25(OH)D is 30–50 ng/mL, with deficiency defined by a circulating level of 25(OH)D < 20 ng/mL [17]. 25(OH)D is further hydroxylated in the kidneys or monocytes by 1α-hydroxylase to create 1-alpha, 25-dihydroxyvitamin D (1,25(OH)2D or calcitriol), the physiologically active form of vitamin D [17,18].

Vitamin D metabolism is tightly regulated by cytochrome P450 (CYP) enzymes. CYP2R1 acts as a major hepatic 25-hydroxylase, catalyzing the conversion of vitamin D to circulating 25(OH)D, whereas CYP27B1 (1α-hydroxylase) converts 25(OH)D into biologically active 1,25(OH)2D. In contrast, CYP24A1 (24-hydroxylase) initiates the catabolism of both 25(OH)D and 1,25(OH)2D, thereby limiting vitamin D signaling. Importantly, CYP27B1 and CYP24A1 are also expressed in extra-renal tissues, including the gastrointestinal tract, where their local expression may regulate tissue-specific availability of active vitamin D. Alterations in these enzymes in digestive neoplasms may therefore modify local vitamin D activity independently of circulating 25(OH)D concentrations [19,20,21].

Vitamin D exerts its effects by binding to the specific vitamin D receptor. VDR is a member of the intracellular nuclear receptor superfamily and is expressed in numerous tissues, including digestive tissues such as the small intestine, colon and pancreas [22]. VDR function as transcription factors, modulated by their ligand, to regulate the expression of genes involved in cell growth, differentiation and apoptosis. VDR can also interfere in these processes through post-transcriptional and post-translational mechanisms [23,24,25,26,27].

In digestive tissues and malignancies, vitamin D/VDR signaling has been implicated in the regulation of inflammation and several cancer-related processes, including cell proliferation and differentiation, apoptosis, angiogenesis, and tumor–microenvironment interactions [13].

In addition, studies have shown that the VDR interacts with other cell signaling pathways, such as the Wnt/β-catenin pathway, which is involved in tumorigenesis [17].

Genetic variability in VDR may also contribute to inter-individual and inter-population differences in vitamin D-related effects. Common VDR polymorphisms include FokI (rs2228570), BsmI (rs1544410), ApaI (rs7975232), and TaqI (rs731236). A systematic review and meta-analysis found that the TaqI variant allele and the FokI FF genotype were associated with a greater response to vitamin D supplementation, whereas no significant associations were observed for BsmI or ApaI [28]. Importantly, the prevalence and cancer-related associations of VDR polymorphisms may vary across ethnic groups, suggesting that genetic background may contribute to population-level heterogeneity in vitamin D-related effects [29]. This variability may also be relevant to digestive malignancies, as a recent meta-analysis of gastrointestinal cancers reported tumor- and region-specific associations between VDR polymorphisms and cancer susceptibility [30]. Overall, VDR genetic variability may partly contribute to the heterogeneous effects associated with vitamin D and raises the possibility of a pharmacogenomic approach to supplementation. VDR genotyping, particularly when considered alongside baseline 25(OH)D status, could potentially help identify individuals more likely to respond to supplementation. At present, however, specific VDR genotypes cannot reliably predict clinical benefit, particularly in patients with digestive malignancies, and prospective genotype-stratified studies are needed before such an approach can be translated into clinical practice.

However, the biological consequences of VDR signaling may differ across digestive malignancies according to tumor type, cellular context, VDR expression, and local vitamin D metabolism. These differences provide the rationale for examining the molecular mechanisms of vitamin D/VDR signaling and their tumor-specific relevance in digestive neoplasms.

## 4. Vitamin D and Carcinogenesis—Molecular Mechanisms

In contrast to its traditional role in controlling calcium and bone metabolism, an increasing number of studies published in the last decade suggest a potential role for vitamin D and its metabolites in the treatment or prevention of several types of cancer [31].

The first evidence suggesting a potential relationship between vitamin D and carcinogenesis dates back to 1980, when Garland and Garland reported an inverse ecological association between solar radiation exposure and colon cancer mortality in the United States and proposed that differences in cutaneous vitamin D synthesis might contribute to this geographical pattern [32].

Subsequent ecological studies investigated associations between solar ultraviolet B (UVB) exposure and the incidence or mortality of a broad range of malignancies, although the strength and consistency of these associations varied according to cancer type and study population [33]. More recent ecological evidence from the United States, based on cancer incidence data from 2016–2020, identified significant inverse associations between solar UVB exposure and the incidence of several malignancies, including esophageal, gastric, and pancreatic cancers [8].

Subsequently, new studies have shown that both vitamin D and its metabolic products can directly inhibit the development and progression of several types of tumors. Frequently, vitamin D deficiency has been associated with an increased risk of colon [34], breast [35] and prostate [36,37] cancer in population studies. Animal studies have also shown that cancer risk is increased by severe vitamin D deficiency [38,39] or by deletion of the gene encoding the vitamin D receptor (VDR) [40,41,42]. In addition, tumor incidence or size has been significantly reduced following administration of vitamin D analogues in experimental models [43,44,45,46,47]. However, the exact mechanisms underlying the potential protective effects of vitamin D in carcinogenesis remain incompletely understood.

The classical genomic mechanism of vitamin D action involves binding of the active metabolite 1,25(OH)_2_D_3_ to VDR, a ligand-activated transcription factor. Ligand-bound VDR preferentially heterodimerizes with retinoid X receptor (RXR), and the VDR–RXR complex recognizes vitamin D response elements (VDREs) within regulatory regions of target genes, thereby modulating their transcription [48,49,50]. However, current evidence indicates that VDR-mediated transcriptional regulation extends beyond this classical model. Genome-wide studies have demonstrated that ligand-activated VDR binds to thousands of genomic loci and functions within a dynamic chromatin environment involving multiple partner proteins and pioneer factors. These interactions are associated with changes in chromatin accessibility and enable the regulation of hundreds of vitamin D target genes. Consequently, vitamin D signaling induces epigenome- and transcriptome-wide changes, with VDR-mediated transcriptional responses strongly influenced by the cellular and chromatin context [51,52].

In addition to transcriptional regulation, other mechanisms of action of vitamin D are mentioned in the literature and will be discussed below. A concise summary of the principal molecular mechanisms through which vitamin D may exert its antitumor effects is provided in Table 1.

### 4.1. Cell Proliferation, Cell Cycle Regulation and Apoptosis

One of the most well-studied roles of vitamin D is growth inhibition on high multiplication rate cells, i.e., tumor cells, but also normal cell types that become tumor cells. Thus a dose-dependent reduction in the rate of multiplication was observed for the first time in melanoma cells treated with 1,25-dihydroxyvitamin D_3_ [53]. Similar effects were subsequently reported in other tumor cell types derived from colon [54], breast [55] and prostate tissues [56]. Also, cell growth was significantly inhibited after 1,25(OH)_2_D_3_ administration in prostate cancer cell lines with VDR overexpression, whereas in the absence of VDR in mouse keratinocytes and osteosarcoma cells 1,25(OH)2 D had no effect [57,58]. Furthermore, increased proliferation rate and reduced apoptosis in prostate epithelial cells after specific deletion of the gene encoding the VDR have been reported, suggesting that vitamin D-mediated growth inhibition is dependent on the presence of the VDR. However, Costa et al. [59] suggest that 1,25(OH)_2_D_3_ may also act through VDR-independent mechanisms since VDR-mediated knockdown of VDR by anti-VDR siRNA did not significantly reduce the growth inhibitory effect of vitamin D.

Potential direct effects of vitamin D on genes involved in cell cycle regulation have also been studied. Thus, 1,25(OH)_2_D_3_ was found to be implicated in the transcriptional regulation of the gene encoding the cyclin-dependent kinase inhibitor p21, causing upregulation of protein 21 but not of p21 mRNA [60]. Furthermore, in primary prostate epithelial cells, suppression of CDK1 mRNA expression was observed 24 h after treatment with 1,25-dihydroxyvitamin D_3_ [1,25(OH)_2_D_3_] [61]. In 2 breast cancer lines, microarray analyses revealed up-regulation of transcription of genes encoding cell cycle inhibitory proteins under the influence of 1,25(OH)_2_D_3_ [62].

In addition to their direct effects on cell proliferation, 1,25(OH)_2_D_3_ and vitamin D analogues may modulate growth factor signaling, including the insulin-like growth factor (IGF) pathway. In prostate cancer cells, 1,25(OH)_2_D_3_ and the synthetic vitamin D analogue EB1089 have been shown to modulate the IGF-II/IGFBP-3 axis [63], while treatment with 1,25(OH)_2_D_3_ increased IGFBP-3 expression in prostate epithelial cells [64]. Microarray analysis of LNCaP prostate cancer cells also identified increased IGFBP3 mRNA expression following treatment with 1,25(OH)_2_D_3_ [65]. Furthermore, Nickerson and Huynh reported that treatment with EB1089 for 14 days was associated with prostate regression and increased prostatic expression of several IGF-binding proteins, including IGFBP-3, in rats [66].

Another way vitamin D is involved in modulating cell proliferation and apoptosis is by interacting with transforming growth factor beta 2 (TGFβ2). TGFβ2 is an antiproliferative factor in both normal epithelial cells and tumor cells, and short-term administration of 1,25(OH)_2_D_3_ or its analogs has been associated with increased TGFβ2 mRNA expression in various cancer cell lines (primary prostate cancer cells, MDA-MB-231, MCF-7, MCF10CA1a) [61,62,67,68]. In addition, 1,25(OH)2 D and its analogs have also been shown to interact with other TGFβ superfamily members such as TGFβ1, GDF15, TGFBR1, SMAD6 and bone morphogenetic proteins, with antiproliferative and tissue morphogenesis-regulating effects [62,65,66,67].

Ultimately, vitamin D mediates cell growth inhibition by blocking the function of β-catenin, a protein involved in the Wnt signaling pathway. Following activation of the Wnt signaling pathway, β-catenin accumulates in the cytoplasm of cells, dissociates from tumor suppressor protein (TSCP) and migrates to the nucleus, where it activates the transcription of genes involved in the synthesis of proteins that control cell proliferation (c-myc and cyclin D1) [69]. In certain colon cancer cell lines, 1,25(OH)2 D can prevent gene transcription by forming a VDR-β-catenin complex [31]. This interaction has also been demonstrated by Shan et al. in HEK293 kidney cells [70]. Furthermore, vitamin D may also act indirectly by increasing the production of E-cadherin, a membrane protein that prevents the accumulation of β-catenin in the nucleus. However, the repression of gene transcription induced by vitamin D treatment even in cells lacking E-cadherin suggests the existence of several mechanisms by which 1,25(OH)2 D may interfere with the Wnt signaling pathway [70]. However, it remains to be seen whether vitamin D can also modulate β-catenin function in other tumor cells except colon and breast.

In addition to antiproliferative effects, there is evidence that 1,25(OH)2 D can also initiate apoptosis, both by increasing the synthesis of pro-apoptotic proteins (Bax and GoS2) and by decreasing the synthesis of anti-apoptotic proteins (Bcl2 and Bcl-XL) in various tumor cell lines such as breast cancer, colorectal cancer, undifferentiated gastric cancer, prostate cancer and chronic myeloid leukemia [71,72,73,74].

### 4.2. Anti-Inflammatory and Immune Boosting Effects

Vitamin D plays an essential role in modulating the immune response and reducing inflammation, critical in the context of digestive neoplasms. Studies have shown that vitamin D regulates the activity of immune cells, including T lymphocytes and macrophages, through its specific receptor, VDR. This receptor is expressed on many types of immune cells, and its activation leads to a decreased production of proinflammatory cytokines and an increase in anti-inflammatory cytokines [75]. In addition, vitamin D influences the intestinal mucosal barrier, helping to maintain its integrity and prevent bacterial transmission and endotoxins, factors that can aggravate inflammation and promote carcinogenesis [76]. Vitamin D deficiency has been associated with an increased risk of development and progression of digestive neoplasms, suggesting that adequate supplementation may have a protective effect [22]. Thus, investigating vitamin D levels and correcting possible deficiencies may be promising therapeutic strategies in the management of patients with digestive neoplasms.

### 4.3. Additional Mechanisms Underlying the Antitumor Effects of Vitamin D

In addition to its effects on cell proliferation, apoptosis, and inflammatory signaling, vitamin D has been implicated in several other cellular processes potentially relevant to its antitumor activity. These include autophagy, antioxidant defense, prostaglandin metabolism, and angiogenesis, which are discussed below.

#### 4.3.1. Autophagy

Autophagy is a process of degradation of cytosolic macromolecules and cell organelles at the lysosomal level, and is generally considered a way to protect cells under stress. However, autophagy can also be used to inhibit tumor growth by killing cancer cells [77].

The first evidence for the involvement of vitamin D in triggering autophagy comes from Mathiasen et al. [78], who found that 1,25(OH)2 D induced growth arrest and cell death by a mechanism independent of caspase and p53. Autophagy induced by a vitamin D analog was subsequently observed in MCF-7S1 cells by Hoyer-Hansen [79]. A pro-autophagic effect of vitamin D has also been reported in HL-60 leukemic cells by suppressing the activity of the anti-autophagic protein mTOR as well as by increasing the level of the pro-autophagic protein beclin-1 [80]. However, currently no in vivo research has been able to directly link autophagy with the anti-tumor actions of vitamin D and its analogs, but this mechanism is in the focus of future research.

#### 4.3.2. DNA Repair and Redox Homeostasis

Oxidative stress-induced DNA damage and impairment of DNA repair mechanisms are important contributors to carcinogenesis [81]. Vitamin D has also been implicated in the regulation of cellular redox homeostasis. Experimental studies indicate that 1,25(OH)_2_D_3_ can modulate antioxidant defense mechanisms and the cellular response to oxidative stress, although these effects may vary according to the cellular context [82,83,84].

Beyond its effects on cellular redox homeostasis, 1,25(OH)_2_D_3_ may contribute to genomic stability by modulating the DNA damage response. Experimental evidence has shown that 1,25(OH)_2_D_3_ induces the expression of the DNA repair genes ATM and RAD50 and promotes DNA double-strand break repair in a VDR-dependent manner. Furthermore, 1,25(OH)_2_D_3_ promoted both major double-strand break repair pathways, homologous recombination and non-homologous end joining, although the effect was more pronounced for homologous recombination [85]. Collectively, these mechanisms may contribute to the antitumor effects associated with vitamin D/VDR signaling.

#### 4.3.3. Prostaglandin Metabolism

Prostaglandins are bioactive lipid mediators involved in inflammatory signaling and tumorigenesis, and their synthesis and degradation are regulated primarily by cyclooxygenases and prostaglandin-degrading enzymes, respectively [86]. Vitamin D signaling has been proposed to modulate this pathway, with evidence relevant to digestive neoplasms deriving particularly from colorectal experimental and clinical studies. In colon cancer cells, 1,25(OH)_2_D_3_ attenuated TNF-α-induced COX-2 expression, although no significant effect on 15-hydroxyprostaglandin dehydrogenase (15-HPGD), the principal prostaglandin-degrading enzyme, was observed under the experimental conditions examined [87]. Consistent with these experimental findings, a placebo-controlled chemoprevention trial in patients with colorectal adenomas showed that vitamin D_3_ supplementation reduced COX-2 expression in morphologically normal colorectal mucosa and significantly decreased the COX-2/15-HPGD expression ratio compared with placebo [88].

#### 4.3.4. Inhibition of Angiogenesis

1,25(OH)2 D can inhibit the development of tumor vasculature, angiogenesis being an essential process for solid tumor progression and tumor cell metastasis. This is due to vitamin D’s ability to reduce proliferation of aortic and tumor-derived aortic endothelial cells, but also to prevent vascular endothelial growth factor (VEGF)-induced endothelial cell elongation [89,90]. This effect of vitamin D has been demonstrated in vivo by Chung et al. who found that VDR knockout mice developed larger prostate tumors with greater vascular volume [91].

Vitamin D may also influence angiogenesis through its action on epithelial cells. The main proposed mechanism is the suppression by 1,25(OH)2 D of the expression of members of the VEGF family, the main pro-angiogenic factor. There is evidence that 1,25(OH)_2_D_3_ reduces VEGF expression in several cancer cell lines [92], while in vivo studies have demonstrated decreased VEGF expression and tumor angiogenesis in experimental colon carcinogenesis [93].This finding suggests that the inhibitory effects of vitamin D on angiogenesis are realized by a mechanism dependent on the presence of HIF-1. In addition, 1, 25(OH)2 D can reduce the mRNA level of the pro-angiogenic cytokine IL-8 in prostate cancer cells by limiting transcription of the IL-8 gene [94].

**Table 1:** Principal molecular mechanisms potentially contributing to the antitumor effects of vitamin D.

Mechanism	Molecular/Cellular Pathways	Effects of Vitamin D (via VDR or Analogs)	Potential Implications for Cancer	References
Cell proliferation, cell-cycle regulation, and apoptosis	Regulation of CDK inhibitors (p21, p27); modulation of Wnt/β-catenin signaling and apoptosis-related pathways	↓ Cell proliferation and cell-cycle progression; ↑ pro-apoptotic signaling; inhibition of β-catenin/TCF transcriptional activity and β-catenin nuclear localization	Growth arrest, induction of apoptosis, and attenuation of proliferative signaling	[72,95,96]
Anti-inflammatory and immune effects	Modulation of NF-κB signaling and cytokine production; VDR-mediated regulation of innate and adaptive immune responses; maintenance of intestinal epithelial barrier integrity	↓ Pro-inflammatory signaling and cytokine production; modulation of immune-cell responses; enhancement of epithelial barrier function	Attenuation of chronic inflammation and modulation of the tumor-associated immune microenvironment	[4,97]
Autophagy	Modulation of mTOR signaling and Beclin-1-dependent autophagy	Inhibition of mTOR signaling; induction of Beclin-1-dependent autophagy and autophagosome formation	Modulation of autophagic responses that may contribute to the antitumor effects of vitamin D	[79,98]
DNA repair and redox homeostasis	Modulation of cellular redox homeostasis and antioxidant defense mechanisms; induction of ATM and RAD50; modulation of DNA double-strand break repair	Modulation of cellular responses to oxidative stress; ↑ ATM and RAD50 expression; promotion of DNA double-strand break repair through homologous recombination and non-homologous end joining	Modulation of oxidative stress responses and maintenance of genomic stability	[82,83,84,85]
Prostaglandin metabolism	Modulation of COX-2 expression and the balance between prostaglandin synthesis and degradation	↓ COX-2 expression; ↓ COX-2/15-HPGD expression ratio	Attenuation of pro-inflammatory, prostaglandin-associated signaling relevant to colorectal carcinogenesis	[87,88]
Inhibition of angiogenesis	Modulation of HIF-1α/VEGF signaling and endothelial cell responses	↓ HIF-1α protein expression and transcriptional activity; ↓ VEGF expression; inhibition of endothelial cell proliferation and tumor-associated angiogenesis	Suppression of tumor vascularization and angiogenic signaling	[91,92]

**Note:** ↑ indicates an increase or upregulation; ↓ indicates a decrease or downregulation.

Although several of these mechanisms have been characterized across different tumor models, their relevance to digestive malignancies is increasingly supported by tumor-specific evidence and is considered below in conjunction with the clinical findings summarized in Section 5.

## 5. Evidence from Preclinical and Clinical Studies on Vitamin D in Digestive Neoplasms

The organization of the data presented in this review was done according to tumor location in the gastrointestinal tract to highlight that each tissue may have unique risk factors and mechanisms for cancer development. Vitamin D receptor density also undergoes organ-dependent changes that may help to establish personalized therapeutic strategies.

However, despite the accumulating evidence of vitamin D’s pleiotropic effects, the translation of preclinical findings into clinical benefit remains challenging. Differences in vitamin D metabolism across gastrointestinal organs, variability in VDR expression and heterogeneity in how vitamin D status is quantified (sun exposure, serum 25[OH]D, dietary intake) introduce substantial confounding. Future studies should harmonize exposure metrics and include intratumoral assessment of VDR, CYP27B1, and CYP24A1 expression to enable more reliable comparisons and clarify whether systemic vitamin D levels accurately reflect local biological activity.

### 5.1. Esophageal Cancer

Esophageal cancer is a relatively rare form of digestive cancer with a low survival rate [99]. Esophageal cancer has 2 subtypes depending on the type of cells involved: squamous cell carcinoma (originating in squamous cells) and esophageal adenocarcinoma (originating in mucus-secreting cells). While excessive alcohol and tobacco consumption appear to greatly increase the risk of developing squamous cell carcinoma, Barrett’s esophagus is the main risk factor associated with esophageal adenocarcinoma [100,101]. Barrett’s esophagus involves metaplasia of the normal esophageal epithelium against a background of hostile environmental factors such as gastro-esophageal reflux disease. Gastric acid hypersecretion combined with reflux disease can trigger an inflammatory response in which keratinocytes in the esophagus release various pro-inflammatory molecules. These molecules are responsible for attracting blood leukocytes, a process that ends with the release of reactive oxygen species [102]. These inflammatory phenomena have been shown to alter the phenotypic expression of genes, including the gene encoding the vitamin D receptor. A study by Trowbridge et al. found that vitamin D receptor expression varies inversely with the differentiation of the esophageal neoplasm. In addition, the same authors reported that neoplastic transformation causes translocation of VDR protein into the cytoplasm [103]. The responsiveness of a cell to vitamin D is dependent on hormonereceptor interaction; this interaction is indispensable for the actions of vitamin D, previously reported in the literature, as a regulator of genes involved in the cell cycle and apoptosis [102].

In addition, based on epidemiological observations, it has been hypothesized that vitamin D status is associated with limiting cell proliferation in breast, prostate and colon neoplasms [102]. This effect of vitamin D could be similar in the esophagus, a claim supported by the result of a recent review, which found that sun exposure decreases the incidence and improves the progression of esophageal cancer [104]. However, studies looking at the association between vitamin D status and esophageal cancer progression using other indicators (e.g., serum vitamin D levels) have reported mixed results [102]. Another mechanism by which vitamin D might be involved in the progression of esophageal cancer involves reduced expression of interleukin-6 in esophageal squamous cells. This hypothesis was described by Chen et al. who found that calcitriol supplementation significantly decreased tumor progression in vivo and in vitro [105].

Furthermore, a recent study found that vitamin D_3_ supplementation reduced the risk of recurrence or death in patients with esophageal squamous cell carcinoma (ESCC) and esophageal adenocarcinoma (EAC) by 73%, with a notable 5-year recurrence-free survival rate of 81% among those taking vitamin D_3_ compared with 31% in the placebo group. However, this effect was not observed in patients with non-p53-immunoreactive tumors [106].

In contrast, a systematic review and meta-analysis by Zgaga et al. found inconsistent evidence regarding the association between markers of vitamin D exposure and esophageal cancer risk [107]. The authors highlighted substantial heterogeneity according to the methods used to assess vitamin D exposure and noted the limited evidence available for the two major histological subtypes of esophageal cancer, underscoring the need for further research to clarify these associations.

More recent vitamin Drelated research has refined these concepts by highlighting both molecular complexity and subtype-specific uncertainty in esophageal cancer. A systematic review and meta-analysis of gastrointestinal malignancies showed that common VDR gene polymorphisms (FokI, TaqI, BsmI and Cdx2) are significantly associated with gastric and colorectal cancer risk, whereas no consistent association was observed for esophageal cancer [30]. This finding suggests that genetic variation in VDR may play a less prominent or more heterogeneous role in esophageal squamous cell carcinoma and esophageal adenocarcinoma than in other digestive tumors.

At the same time, emerging data on vitamin D–regulated non-coding RNAs indicate that 1,25-dihydroxyvitamin D can markedly reshape transcriptional programs in squamous carcinoma cells. In this context, vitamin D signaling appears to shift from classical transcription factor–driven regulation toward long non-coding RNAs that are largely VDR-dependent and display context-specific antitumor effects [108]. In parallel, narrative and experimental studies in esophageal squamous cell carcinoma emphasize the central role of dysregulated long non-coding RNAs and microRNAs in promoting proliferation, invasion, immune evasion and resistance to therapy, and propose these molecules as promising diagnostic and therapeutic targets [109].

Although these non-coding RNA signatures have not yet been systematically integrated with vitamin D signaling in esophageal tumors, converging evidence from squamous cell models and other gastrointestinal cancers raises the possibility that vitamin D may exert part of its antineoplastic effects through epigenetic modulation of non-coding RNA networks and the tumor microenvironment, rather than through VDR expression alone. Overall, current evidence supports a potential adjuvant role of adequate vitamin D status in digestive oncology, while also underscoring the lack of robust prospective data in esophageal cancer. Future studies should therefore distinguish between esophageal squamous cell carcinoma and adenocarcinoma, consider VDR genetic background, and explore vitamin D–non-coding RNA interactions as novel prognostic and therapeutic avenues.

### 5.2. Gastric Cancer

Gastric cancer is the fourth most common cancer worldwide and it can develop from any part of the stomach [1]. It is often diagnosed in advanced stages and has a poor prognosis. The most important risk factors for gastric cancer are chronic Helicobacter pylori infection, excessive alcohol and tobacco consumption [110].

There is now evidence that serum vitamin D levels are associated with decreased risk of developing gastric cancer, with vitamin D deficiency playing an important role in the process of tumorigenesis [111]. In gastric cancer, vitamin D and its analogues also appear to suppress cancer cell proliferation by regulating the cell cycle and substantially inducing apoptosis in undifferentiated malignant gastric cells [112]. Vitamin D has also been observed to increase VDR and CYP24A1 expression in gastric malignant cells and to interfere with the hedgehog signalling pathway, reducing cell viability by inhibiting key genes [110].

The anti-tumor effects of vitamin D in gastric cancer have also been demonstrated by combining it with anticancer drugs such as vinblastine, paclitaxel and adriamycin [113]. A retrospective study by Ren et al. reported an inverse proportional relationship between serum 25-hydroxyvitamin D [25(OH)D] levels and lymph metastasis [112]. Furthermore, survival rates in patients diagnosed with gastric cancer were found to be better in patients with high serum vitamin D levels compared to patients with low vitamin D levels. However, these results are in contradiction with the study by Khayatzadeh et al. who found no statistically significant correlation between vitamin D intake or serum vitamin D levels and gastric cancer risk [114]. The latter study is also supported by the reports of Giovannucci et al. who also found that the relationship between vitamin D levels and gastric cancer was insignificant [115].

Finally, 24R,25-dihydroxyvitamin D_3_ [24R,25(OH)_2_D_3_] was shown to exert chemopreventive effects in rats with glandular stomach carcinogenesis, reducing the development of cancerous and precancerous gastric lesions [116]. Additional studies indicate an indirect role of vitamin D in gastric cancer through the induction of microRNA species (e.g., miR-99b) capable of inhibiting tumor cell viability by suppressing overexpressed genes in tumor cell lines (Hox D3) [117]. In addition, the eradication rate of Helicobacter pylori infection, the main risk factor for gastric cancer, appears to be dependent on serum vitamin D levels. Yildirim et al. have shown that vitamin D deficiency contributes to the decreased eradication rate of Helicobacter pylori infection, along with other factors such as antibiotic resistance of the microorganism and genetic disorders of the host organism [118]. Moreover, a recent study revealed that patients with p53-immunoreactive tumors who received vitamin D had a significantly higher rate of relapse-free survival at five years compared to those who received a placebo [106].

However, despite the large amount of data supporting the antitumor effects of vitamin D in gastric cancer (Table 2), the precise mechanism by which it acts is not yet fully understood. Also, recommendations on the optimal dose of vitamin D that would result in maximum antitumor effects have not been established, nor has the influence of vitamin D in different stages of gastric cancer.

The discordance between positive preclinical findings and conflicting epidemiological results suggests that serum vitamin D may be a surrogate marker of overall health rather than a direct mediator of tumor suppression. Geographic variation in Helicobacter pylori prevalence, genetic polymorphisms in VDR, and stage-specific differences in CYP24A1 overexpression could all modulate the biological response to vitamin D. Moreover, the dual observation of increased VDR and CYP24A1 in tumor tissue raises the possibility that local vitamin D inactivation limits therapeutic efficacy, even when systemic levels are adequate. Randomized trials with standardized dosing and concurrent assessment of intratumoral vitamin D metabolism are needed to define any true causal role.

### 5.3. Liver Cancer

Liver tumors are often metastases of cancers that originally developed in other organs. Primary liver cancers are less common and fall into 2 subtypes: hepatocellular carcinoma and cholangiocarcinoma. The fact is that both types of liver cancer develop on a pre-existing pathological substrate [119,120].

Current scientific evidence points to a protective role of vitamin D in liver cancer patients by reducing cell proliferation in malignant cell lines. Dysregulation of vitamin D metabolism has also been described in liver cancer, including altered expression of vitamin D-metabolizing enzymes. In HCC cell lines, 1,25(OH)_2_D_3_ induced a marked increase in CYP24A1 expression in a cell line-dependent manner [121,122]. Vitamin D also possesses an antifibrotic effect in liver tumor cells by modulating collagen synthesis and the metallopeptidase responsible for collagen degradation. A VDR-mediated cytostatic effect of vitamin D and its synthetic analogues has also been described, by inducing apoptosis, in liver tumor cells expressing this receptor [123]. Regarding the role of vitamin D in the progression of liver cancer, Finkelmeier et al. showed that severe 25(OH)D deficiency was associated with higher mortality in patients with hepatocellular carcinoma, and circulating 25(OH)D concentrations were independently associated with the risk of death [124]. Seubwai et al. also support the hypothesis that vitamin D may be an important therapeutic factor by reporting a positive response of VDR-expressing malignant liver cell lines to vitamin D treatment [125].

However, the role of vitamin D in the progression of liver cancer remains questionable and further studies are needed to elucidate precisely the mechanisms underlying the anti-proliferative effects of vitamin D in malignant cell lines. The extent to which the level of VDR expression in different malignant cell lines influences the response to vitamin D treatment should also be explored. In addition, it is interesting to study whether severe vitamin D deficiency in liver cancer is due to overexpression of CYP24A1 in tumor cells, or whether this deficiency is correlated with advanced liver dysfunction with reduced synthesis of vitamin D transport protein and hydroxylation processes of vitamin D precursors. Because the liver is central to vitamin D activation and transport, low circulating vitamin D in hepatocellular carcinoma may reflect hepatic failure rather than tumor-driven depletion. Overexpression of CYP24A1 could represent either an adaptive response to high ligand levels or a mechanism of tumor resistance. Disentangling these processes will require simultaneous measurement of systemic 25(OH)D, active 1,25(OH)2D, hepatic synthetic capacity and intratumoral enzyme activity in prospective clinical cohorts.

### 5.4. Pancreatic Cancer

The association between vitamin D status and pancreatic cancer risk has been investigated in several cohort studies [126,127,128]. Thus, it has been found that pancreatic tumor cells show a deregulation of the vitamin D circuit. An in vitro study shows that the VDR gene is expressed in both cell lines, but pancreatic cancer cells show an increased rate of VDR expression compared to normal pancreatic cells. Also, numerous studies mention the inhibitory effect of vitamin D and its analogues on the proliferation rate of pancreatic malignant tissue [129,130,131]. This hypothesis is based on the ability of calcitriol to bind to sites belonging to signalling pathways involved in the development of pancreatic cancer. These signalling pathways have been extensively described by Jones et al. based on genetic analysis [132]. The main antitumoral effects of calcitriol and its analogues are exerted by cell cycle arrest at the G1/S point, induction of caspase-dependent apoptosis in malignant cells and limitation of tumor migration and invasion.

Epidemiological studies have also shown that vitamin D intake can influence both the incidence and prognosis of pancreatic cancer. Thus, Cho et al. reported that vitamin D deficiency resulted in a poor prognosis in stage III and IV pancreatic cancer patients [133]. However, Piper et al. reported that very high serum 25(OH)D concentrations (≥100 nmol/L) were associated with an increased risk of pancreatic cancer compared with concentrations of 50–<75 nmol/L, providing no support for a uniformly protective association between vitamin D status and pancreatic cancer risk [134]. It should be taken into account that the inconsistency of these results may derive from factors such as the moment in the course of the disease at which vitamin D supplementation occurred and the time interval between vitamin D administration and blood sampling.

Vitamin D has also been proposed as a potential biological link between diabetes and cancer, although its specific contribution to pancreatic cancer in patients with diabetes remains uncertain [135]. Given the antitumoral effects of vitamin D, the association of vitamin D with chemotherapy in pancreatic cancer therapy is also interesting to explore, with current data in the literature being encouraging (Table 2), though insufficient.

To this end, Sherman et al. demonstrated that pharmacological activation of VDR using calcipotriol can reprogram pancreatic stellate cells toward a more quiescent state, thereby remodeling the tumor microenvironment and improving the response to chemotherapy in preclinical models. In mice, the combination of calcipotriol with gemcitabine increased intratumoral chemotherapy concentrations and prolonged survival compared with chemotherapy alone [136].

Nevertheless, epidemiologic studies remain inconsistent, with some paradoxically suggesting higher serum vitamin D levels associate with increased risk. These discrepancies may stem from reverse causation, differences in disease stage at sampling, or effects of malabsorption and cachexia on vitamin D kinetics. While stromal reprogramming by synthetic VDR ligands is a promising avenue to enhance chemotherapy delivery, translation from murine models to human disease will require careful dose optimization, toxicity monitoring and biomarker-driven patient selection.

### 5.5. Colorectal Cancer

Colorectal cancer is the second leading cause of cancer mortality worldwide and is more common in developed than in underdeveloped regions [137]. In recent decades, numerous risk factors for colorectal cancer have been described, the most studied being: age, obesity, interaction with environmental factors (chemical pollutants, infectious agents, radiation) and genetic component (mutations, immune system dysfunction, hormonal disorders). There is also strong evidence regarding the role of diet in the development of colorectal cancer, with excessive red meat consumption and, high calorie and low fiber intake being associated with a poor prognosis [138,139]. Vitamin D status has also been investigated in relation to colorectal neoplasia, with higher circulating 25(OH)D concentrations being associated with a lower risk of colorectal adenoma [140].

Vitamin D acts in colorectal epithelial cells as a regulator of cell cycle, proliferation, differentiation and apoptosis by binding to the specific receptor VDR. Overexpression of the VDR gene in colorectal cancer has been shown to have a direct association with PIK3CA and KRAS mutations [141]. It has also been reported that vitamin D induces the expression of cyclin-dependent kinase inhibitors such as p21 and p27, which are able to limit tumor growth by suppressing proto-oncogenes such as c-myc and cyclin-D1 [142]. Furthermore, Vitamin D has also been shown to induce the antimicrobial peptide cathelicidin in human colonic epithelial cells, although this evidence derives primarily from inflammatory rather than neoplastic models [143].

Observational studies and meta-analyses have reported associations between circulating 25(OH)D concentrations and colorectal cancer risk, colorectal adenoma, and clinical outcomes, although the magnitude and consistency of these associations vary across studies [144,145,146,147,148]. Nevertheless, the available evidence remains heterogeneous, with differences reported across study populations, outcomes, and methodological approaches [146,148].

More recently, Thet et al. conducted a systematic review and meta-analysis evaluating the prognostic significance of circulating vitamin D status in colorectal cancer. The review included 61 studies, of which 35 contributed to the meta-analysis, and showed that higher circulating 25(OH)D concentrations were associated with lower colorectal cancer-specific mortality (HR 0.74, 95% CI 0.69–0.80), lower overall mortality (HR 0.68, 95% CI 0.64–0.72), and improved disease-free survival (HR 0.71, 95% CI 0.61–0.83) [149]. Although these findings strengthen the evidence supporting the prognostic relevance of circulating 25(OH)D in colorectal cancer, they do not establish that vitamin D supplementation itself confers a survival benefit.

Colorectal cancer incidence also appears to be associated with circulating 25(OH)D concentrations. In a large pooled analysis, circulating 25(OH)D concentrations <30 nmol/L were associated with a 31% higher risk of colorectal cancer compared with concentrations of 50–<62.5 nmol/L [150]. However, there are also studies that either contradict this hypothesis or partially support it by finding a positive correlation between serum vitamin D levels and colorectal cancer incidence in women but not in men [146], and in European and American but not in Asian populations [148]. For example, following daily supplementation with calcium and vitamin D over a period of 3–5 years, no significant reduction was found in colorectal adenoma recurrence or in the incidence of all cancers, including colorectal cancer [151,152]. However, a subsequent genetic analysis of the adenoma prevention trial showed that the effect of vitamin D_3_ supplementation on advanced adenoma recurrence differed according to VDR genotype. Among participants with the rs7968585 AA genotype, representing approximately 26% of the analyzed cohort, vitamin D_3_ supplementation was associated with a 64% lower risk of advanced adenomas (RR 0.36, 95% CI 0.19–0.69), whereas this protective association was not observed among carriers of the G allele [153]. These findings suggest that VDR genetic variability may modify the chemopreventive response to vitamin D_3_ and could partly explain why an overall benefit was not observed in the unselected study population. Also, serum 25(OH)D concentration was not related to colorectal adenoma recurrence in a study by Jacobs et al. [154]. Interestingly, although increased consumption of red meat is associated with increased incidence and progression of colorectal cancer, serum vitamin D levels were higher in meat eaters compared to non-consumers [155,156,157]. This finding virtually negates the role of vitamin D in colorectal cancer progression, but it is necessary to study other variables that could influence this result, for example, the polymorphism of the VDR gene. Another study claims that there is a correlation between physical activity level and improved colorectal cancer prognosis [158], but it should be investigated whether the beneficial effects of physical activity in this case do not also include increased sun exposure with a consequent increase in vitamin D levels.

Overall, the colorectal cancer field illustrates the complexity of vitamin D research: large observational studies consistently show lower mortality among individuals with higher vitamin D levels, yet randomized supplementation trials have failed to produce significant preventive effects. These divergent outcomes suggest that vitamin D may act more as a biomarker of a healthier lifestyle, greater outdoor activity, better diet, lower inflammation, than as an independent causal factor. Future interventional studies should stratify by baseline deficiency, genetic polymorphisms and tumor molecular subtype to clarify whether vitamin D supplementation provides benefit beyond correcting deficiency.

**Table 2:** Summary of key experimental and clinical evidence regarding vitamin D in digestive neoplasms.

Cancer Type	Study Type	Main Findings	References
Mixed digestive tract cancers	Post hoc analysis of a randomized clinical trial	In the AMATERASU trial, vitamin D_3_ supplementation was associated with a lower risk of relapse or death in the p53-immunoreactive subgroup of patients with digestive tract cancers; this subgroup finding was derived from a post hoc analysis.	[106]
Esophageal cancer	Experimental study	VDR expression in esophageal adenocarcinoma was associated with tumor differentiation and response-related clinicopathological features.	[103]
	Experimental study	1α,25-Dihydroxyvitamin D3 inhibited esophageal squamous cell carcinoma progression by suppressing IL-6 signaling.	[105]
	Systematic review and meta-analysis	Evidence regarding markers of vitamin D exposure and esophageal cancer risk was inconsistent, with substantial heterogeneity across exposure measures and limited subtype-specific evidence.	[107]
	Systematic review and meta-analysis	Common VDR polymorphisms showed associations with some gastrointestinal cancers, whereas no consistent association was identified for esophageal cancer.	[30]
Gastric cancer	Retrospective study	Lower serum 25(OH)D concentrations were associated with adverse clinicopathological features, including lymph-node metastasis, and with poorer prognosis in patients with gastric cancer.	[112]
	Experimental study	Vitamin D3 reduced cell viability in gastric cancer cells; the study also demonstrated vitamin D-related effects in cholangiocarcinoma cells.	[113]
	Systematic review and meta-analysis	No statistically significant association was identified between vitamin D intake or circulating vitamin D concentrations and gastric cancer risk.	[114]
	Experimental study	Vitamin D3 induced miR-99b-3p, which contributed to antiproliferative effects in gastric cancer cells through targeting of HoxD3.	[117]
Hepatobiliary cancer	Experimental study	Dysregulation of vitamin D3 synthesis was associated with enhanced cholangiocarcinoma growth, supporting a role for local vitamin D metabolism in tumor biology.	[121]
	Experimental study	Vitamin D administration markedly increased CYP24A1 mRNA expression in hepatocellular carcinoma cell lines, indicating active regulation of vitamin D catabolism in these cells.	[122]
	Prospective cohort study	Severe 25(OH)D deficiency was independently associated with poorer prognosis and increased mortality in patients with hepatocellular carcinoma.	[124]
	Experimental and clinicopathological study	VDR overexpression was associated with favorable prognosis in cholangiocarcinoma, and VDR-expressing cholangiocarcinoma cells showed responsiveness to vitamin D-related treatment.	[125]
Pancreatic cancer	Experimental studies	Vitamin D_3_ and vitamin D analogues, including maxacalcitol, inhibited proliferation or reduced cell number in pancreatic cancer cell lines.	[129,130,131]
	Observational study	Vitamin D deficiency was associated with poorer prognosis in patients with pancreatic adenocarcinoma.	[133]
	Nested case-control study	Serum 25(OH)D concentrations ≥100 nmol/L were associated with a significantly higher risk of pancreatic cancer compared with concentrations of 50–<75 nmol/L (OR 3.23, 95% CI 1.24–8.44); overall, the findings did not support a protective association between serum 25(OH)D and pancreatic cancer risk.	[134]
	Preclinical study	Pharmacological VDR activation with calcipotriol reprogrammed pancreatic stellate cells and enhanced the response to gemcitabine in preclinical models.	[136]
Colorectal cancer	Observational molecular-pathology study	VDR expression in colorectal cancer was significantly associated with PIK3CA and KRAS mutations.	[141]
	Systematic reviews and meta-analyses	Higher circulating 25(OH)D concentrations were associated with better overall and colorectal cancer-specific survival.	[147,149]
	International pooled analysis of 17 cohorts	Circulating 25(OH)D <30 nmol/L was associated with a 31% higher colorectal cancer risk compared with 50–<62.5 nmol/L.	[150]
	Randomized trials/trial reanalysis	Calcium and vitamin D supplementation did not provide consistent evidence of colorectal cancer prevention or reduction in colorectal adenoma recurrence in the evaluated trial settings.	[151,152]

To conclude, in this article we have reviewed the role of vitamin D in preventing and improving the prognosis of gastrointestinal neoplasms. Thus, current literature data suggest that the beneficial effects of vitamin D stem from its involvement in several cellular mechanisms such as: cell cycle arrest, inhibition of tumor cell proliferation and differentiation, limitation of invasion and metastasis, induction of apoptosis and inhibition of angiogenesis. The synergistic action of vitamin D with some chemotherapies used in the therapy of digestive neoplasms has also been observed, but it remains to be seen how this dose can be personalized for each patient. Circulating 25(OH)D concentrations may also represent a potential biomarker in gastrointestinal malignancies, but should be interpreted in conjunction with other factors such as sun exposure and dietary intake. The potential contribution of sedentary lifestyles, reduced time spent outdoors, and consumption of highly processed foods to the risk of digestive cancers also warrants further investigation.

## 6. Future Research Directions and Unresolved Questions

**Figure 2:**
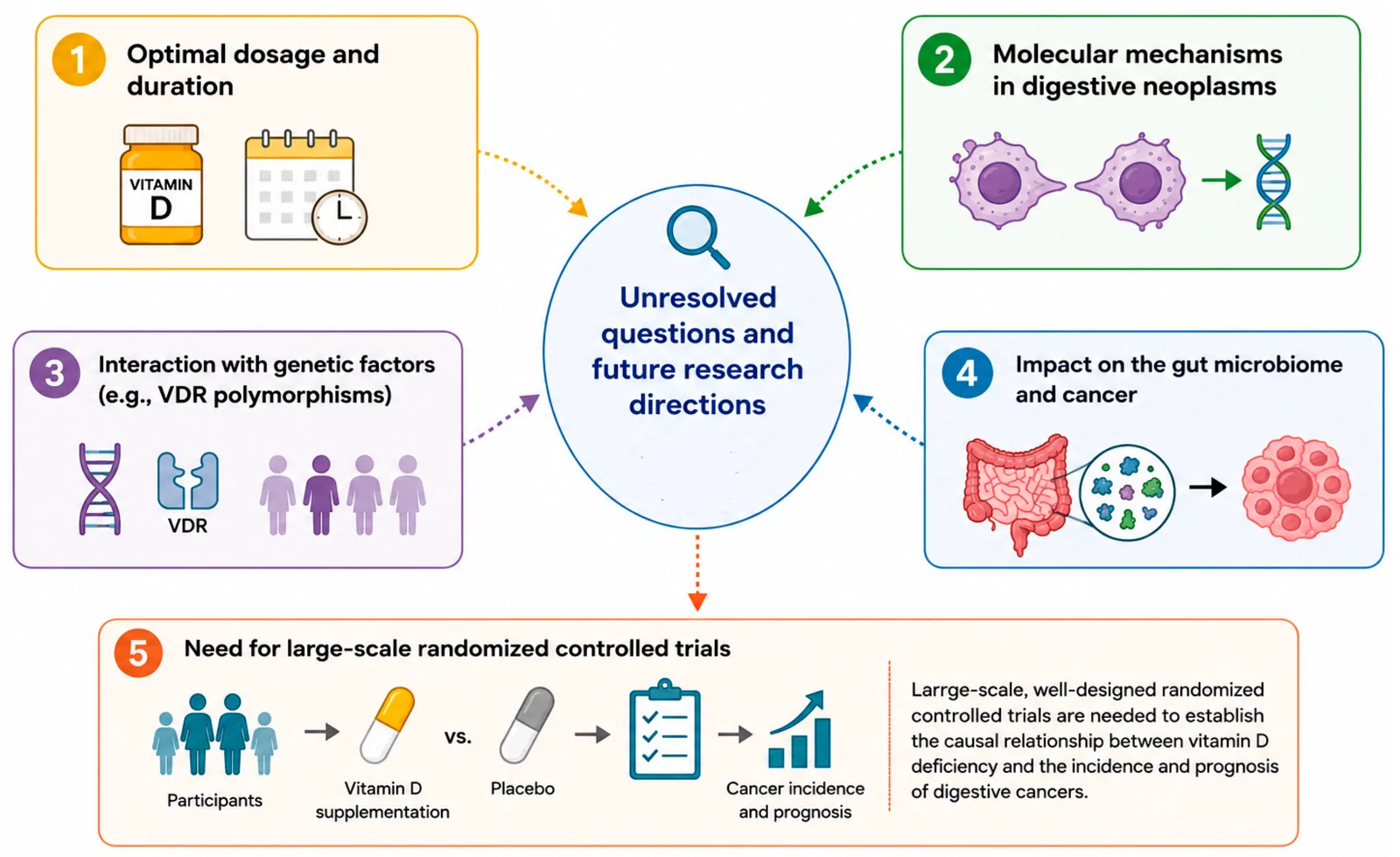
Unresolved questions and future research directions on the role of vitamin D in digestive neoplasms.

While significant progress has been made in understanding the role of vitamin D in the progression of digestive neoplasms, several areas remain unclear and warrant further investigation (Figure 2). One major area of uncertainty is the optimal dosage and duration of vitamin D supplementation necessary to achieve protective effects against these cancers. Additionally, the specific mechanisms by which vitamin D influences cancer cell growth, differentiation, and apoptosis in various types of digestive neoplasms are not fully understood. The interaction between vitamin D levels and genetic factors, such as polymorphisms in the vitamin D receptor (VDR), also remains inconclusive and requires more research. Beyond host genetic variability, tumor-related biomarkers may also influence the response to vitamin D supplementation. In a post hoc analysis of the AMATERASU randomized clinical trial, Kanno et al. found that postoperative vitamin D_3_ supplementation (2000 IU/day) was associated with a significantly lower risk of relapse or death in patients with p53-immunoreactive digestive tract cancer (HR 0.27, 95% CI 0.11–0.61), whereas no benefit was observed in the non-p53-immunoreactive subgroup (HR 1.09, 95% CI 0.65–1.84; P for interaction = 0.005) [106]. These findings suggest that p53 immunoreactivity may represent a potential biomarker for identifying patients more likely to benefit from vitamin D_3_ supplementation, although prospective validation is required before a biomarker-guided approach can be implemented clinically. Furthermore, the impact of vitamin D on the gut microbiome and its potential role in modulating cancer risk and progression is an emerging field that needs more exploration. Finally, large-scale randomized controlled trials incorporating baseline vitamin D status and relevant genetic and tumor biomarkers are needed to determine whether vitamin D supplementation can improve clinically meaningful outcomes in selected patients with digestive cancers.

## 7. Conclusions

The growing body of evidence highlights the complex role of vitamin D in the development and progression of digestive neoplasms. As summarized in Table 2, the VDR-mediated antitumor effects of vitamin D support the biological relevance of the vitamin D–VDR axis in digestive carcinogenesis. Although preclinical studies provide compelling mechanistic evidence, findings from observational and clinical studies remain heterogeneous and do not establish vitamin D supplementation as a stand-alone anticancer therapy. Overall, current evidence suggests that vitamin D status may be relevant to the risk and clinical course of digestive cancers, while its therapeutic significance remains to be established.

## Data Availability

No new data were created or analyzed in this study. Data sharing is not applicable to this article.
